# A Case Report on Clotrimazole-Induced Localized Erythema

**DOI:** 10.7759/cureus.52682

**Published:** 2024-01-21

**Authors:** Indrani Sarma, Namit Ray, Priyotosh Banerjee, Subhadeep Das, Dhriti Kumar Brahma

**Affiliations:** 1 Pharmacology, All India Institute of Medical Sciences, Guwahati, Guwahati, IND; 2 Pharmacology, North Eastern Indira Gandhi Regional Institute of Health and Medical Sciences (NEIGRIHMS), Shillong, IND; 3 Otorhinolaryngology, North Eastern Indira Gandhi Regional Institute of Health and Medical Sciences (NEIGRIHMS), Shillong, IND

**Keywords:** pruritis, ear drop, erythema, clotrimazole, allergy

## Abstract

Clotrimazole, a commonly used antifungal agent, is employed in the treatment of otomycosis and other ear infections. However, its use can lead to adverse drug reactions (ADRs), occasionally manifesting as local irritation or allergic responses. This abstract presents a case study of a patient exhibiting an adverse reaction to clotrimazole ear drops, highlighting the clinical presentation, management, and resolution of the ADR. The patient, a 73-year-old male, presented with itching and erythema over the left pinna extending up to the middle of the sternum following clotrimazole ear drop application. Immediate cessation of the medication and symptomatic treatment led to rapid resolution of symptoms. This case emphasizes the importance of recognizing potential ADRs associated with clotrimazole ear drops and the necessity of prompt intervention to mitigate adverse effects, thereby ensuring optimal patient care.

## Introduction

An adverse drug reaction is a “response to a drug which is noxious and unintended and occurs at doses normally used in man for prophylaxis, diagnosis, or therapy of disease or for the modification of physiologic function" [1]. Topical application of drugs is associated with very less systemic side effects. However, topical application may sometimes be associated with rashes, pruritis, eczema, etc., which are less severe in nature [2].

Clotrimazole is a broad-spectrum antimycotic or antifungal agent. Its antimycotic properties were discovered in the late 1960s [3]. Clotrimazole falls under the imidazole category of azole antifungals. It is available in various preparations, including creams, pessaries, and troche formulations. The minimal side effect profile of this drug and its uncomplicated metabolic profile have led it to gain widespread acceptance for the treatment of mycotic outbreaks, such as vaginal yeast infections [3].

Clotrimazole is also very commonly prescribed for fungal infections in the ear as in otitis externa in the form of topical ear drops [4]. One such fungal infection of the ear is called otomycosis and can be caused in a case of otitis externa. Otitis externa is a term used for inflammation in the ear when it is confined to the ear canal and does not go further than the eardrum. If we then scratch or poke the ear, this can damage the skin in the ear canal and cause inflammation and further infection by bacteria or fungi can occur [5]. Though any severe adverse effects are rare from topical clotrimazole, we have some reported cases of clotrimazole-induced pruritis. Here, however, we discuss a case of localized erythema and pruritis in an elderly patient who subsided on cessation of the medication.

## Case presentation

**Figure 1:**
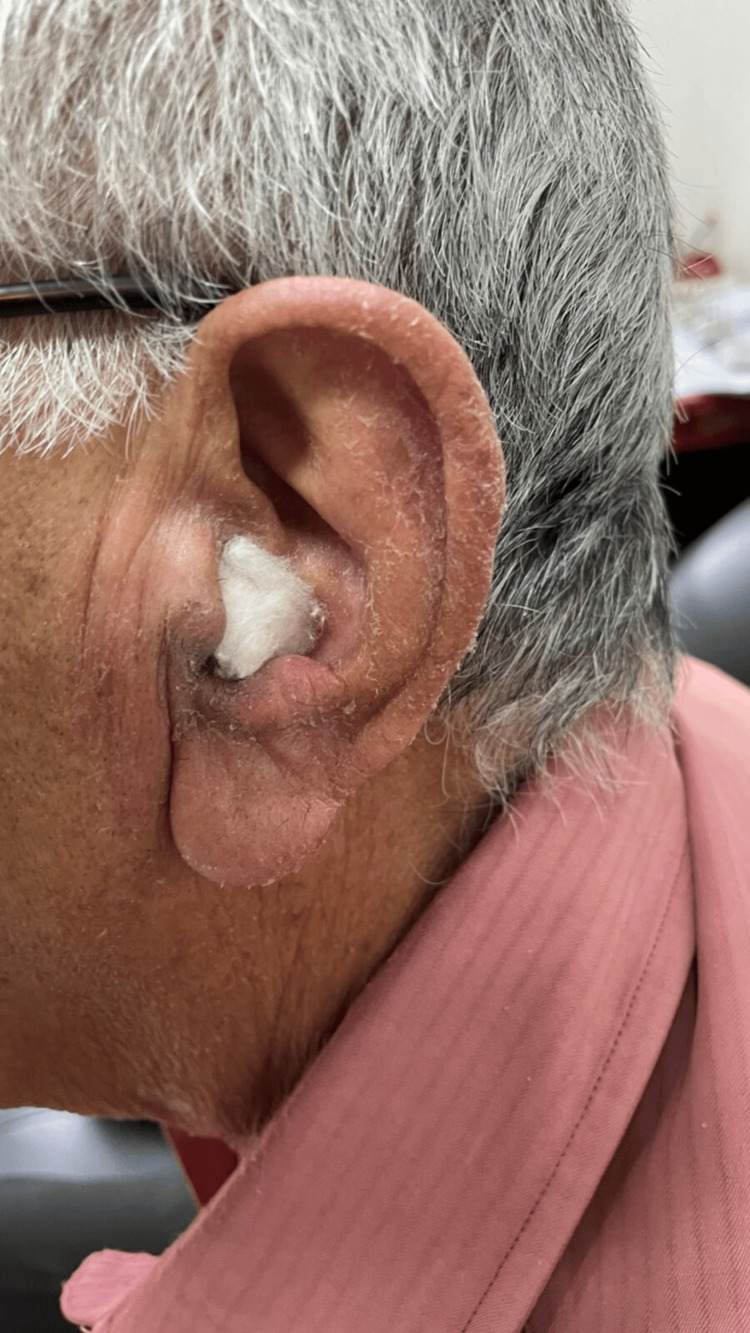
Drying and peeling of the skin over the left pinna

An elderly male of 73 years reported to the outpatient department of our tertiary care center complaining of itching over the left pinna, and this itching extended on the left side up to the margin of his sternum. On examination, there was redness and itching along with a local rise in temperature. The patient was a diagnosed case of otomycosis and had been advised of clotrimazole ear drops for the same. He had been advised to use the drops four times a day. However, application for the first time only resulted in the development of rashes of the pinna, and the dripping of the ear drops over the sternum caused rashes over the chest area. There was dryness and scaling of the skin in the affected area (Figure 1).

**Figure 2:**
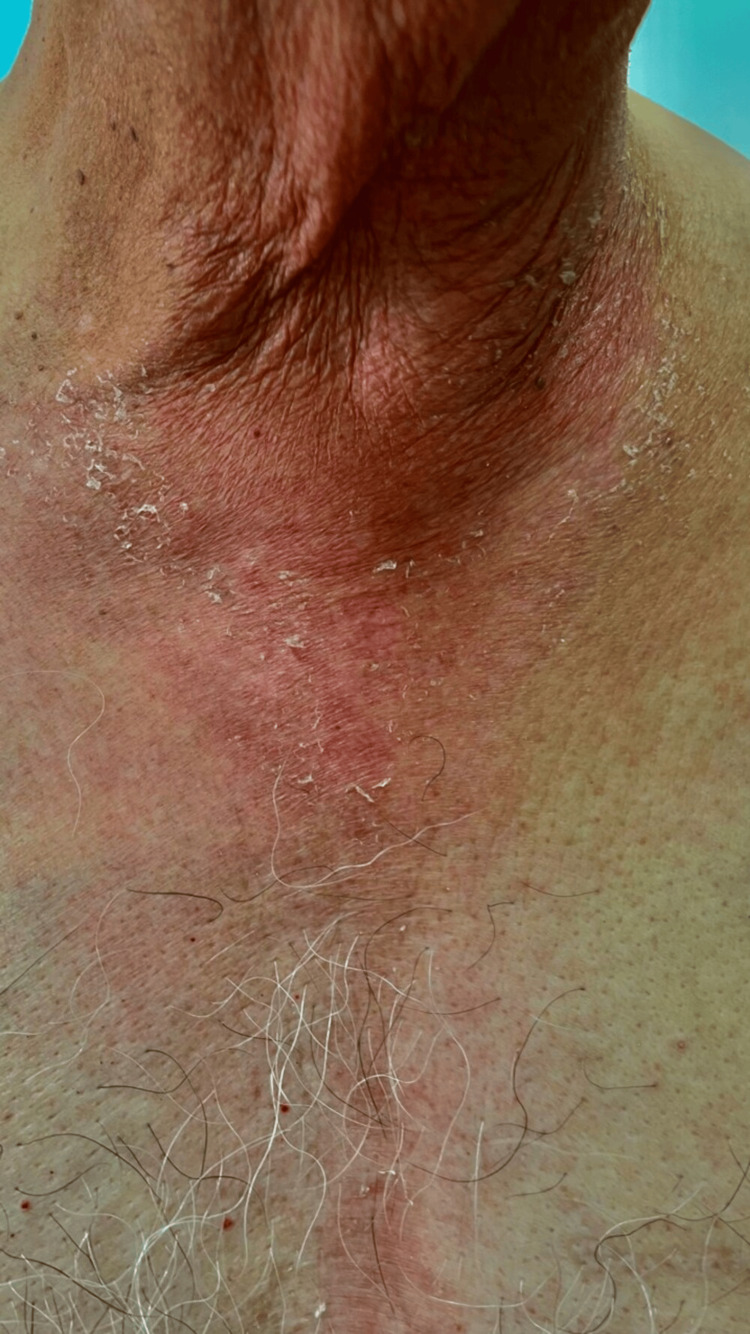
Redness and scaling over the chest up to the sternum

He complained that the symptoms started after using clotrimazole ear drops in the left ear. According to him, the rashes and itching occurred about five minutes after the application of the ear drops and persisted throughout the treatment period until the drug was stopped. The patient also gave a history of otomycosis infection a few days back for which he was ordered the drops for topical application in a dose of two drops four times daily. He was advised of other standard treatments for otomycosis. However, on using the drops, he started developing redness of the pinna along with itching. The consequent dosing led to the spread of the rash from the pinna up to the sternum and chest on the left side as the drops dripped from the ear to the chest (Figure 2).

There was a local rise in temperature but no associated fever. On arrival, the patient was advised to stop the medication and given supportive treatment. By the next day, the rash had subsided, and there was little-to-no pruritis in the affected area.

The patient, though elderly, had no significant history of any other disease and was also not on any concomitant medications. The rash was completely resolved within 48 hours, as confirmed by routine follow-up.

## Discussion

Localized erythema is a rare adverse effect in patients treated with clotrimazole, and there is no evidence of clotrimazole ear drop causing localized erythema as found in this case. The exact mechanism by which clotrimazole ear drops induce localized erythema is not well-understood. However, it is hypothesized that the active ingredient clotrimazole may cause a local allergic reaction or irritation in some individuals [6]. Further research is needed to fully understand the underlying causes of clotrimazole ear drop-induced localized erythema and to identify potential risk factors or patient populations that may be more prone to experiencing this adverse effect. In clinical practice, localized erythema following the use of clotrimazole ear drops is generally a mild and self-limiting side effect. However, without proper knowledge of the same, it becomes difficult for clinicians to act promptly in the case of the occurrence of adverse effects.

If an elderly individual is experiencing localized erythema in or around the ears after using ear drops, several factors could be contributing to this reaction. Factors such as skin thinning due to age and accidental overuse can cause such conditions. Hence, it becomes important to keep an eye out for reactions in the elderly as the manifestations of the effects can increase manifold. Elderly individuals may experience slower healing processes, and localized erythema may persist longer than expected. Thus, getting ahead in the case of management of localized erythema becomes imperative.

There was a strong probable reaction between the drug and the reaction based on the causality assessment done by the WHO-UMC Scale and Naranjo Scale [7,8]. The assessment is included in Tables 1-2.

**Table 1:** Causality assessment by the WHO-UMC Scale Based on these criteria, the causality assessment category is- probable [7]

WHO-UMC Causality Assessment Criteria	
Event or laboratory test abnormality, with a reasonable time relationship to drug intake	Yes
Unlikely to be attributed to disease or other drugs	Yes
Response to withdrawal clinically reasonable	Yes
Rechallenge not required	Not Required

**Table 2:** Causality assessment by the Naranjo Scale Based on the score, the association between the drug and the reaction is probable [8].

Question	Yes	No	Do Not Know	Score
1. Are there previous conclusive reports on this reaction?	+1	0	0	1
2. Did the adverse event appear after the suspected drug was administered?	+2	-1	0	2
3. Did the adverse event improve when the drug was discontinued or a specific antagonist was administered?	+1	0	0	1
4. Did the adverse event reappear when the drug was readministered?	+2	-1	0	0
5. Are there alternative causes that could on their own have caused the reaction?	-1	+2	0	2
6. Did the reaction reappear when a placebo was given?	-1	+1	0	0
7. Was the drug detected in blood or other fluids in concentrations known to be toxic?	+1	0	0	0
8. Was the reaction more severe when the dose was increased or less severe when the dose was decreased?	+1	0	0	0
9. Did the patient have a similar reaction to the same or similar drugs in any previous exposure?	+1	0	0	0
10. Was the adverse event confirmed by any objective evidence?	+1	0	0	1
			Total Score: 7	

## Conclusions

Though clotrimazole is a relatively safe and effective drug, especially when used as a topical ear drop, further studies are required to establish a definitive relationship between the localized erythema and the drug itself, as well as its relation to the age of the patient. As this reaction occurs in the elderly, it becomes very difficult for management in such cases as most of these patients are often not very compliant. The pathway of clotrimazole leading to localized erythema also needs to be studied further. Early detection and prompt treatment become essential to prevent further spread of the rash.
